# *‘Making the invisible visible’* through alcohol screening and brief intervention in community pharmacies: an Australian feasibility study

**DOI:** 10.1186/s12889-016-3805-3

**Published:** 2016-11-08

**Authors:** H. Laetitia Hattingh, Jonathan Hallett, Robert J. Tait

**Affiliations:** 1 School of Pharmacy, Curtin University, Kent Street, Bentley, Australia; 2 School of Public Health, Curtin University, Kent Street, Bentley, Australia; 3 National Drug Research Institute, Faculty of Health Sciences, Curtin University, 10 Selby Street, Shenton Park, Australia

**Keywords:** Alcohol brief intervention, Community pharmacy, Screening, AUDIT tool

## Abstract

**Background:**

Screening and brief interventions (SBI) for alcohol related problems have been shown to be effective in health settings such as general practice or emergency departments. Recent data from the United Kingdom and New Zealand suggest that SBI can be delivered through community pharmacies, but this approach has not been tested in Australia. This study assesses the feasibility of delivering alcohol SBI via community pharmacists.

**Method:**

We recruited five pharmacies and developed an SBI training package to be delivered by pharmacy staff, who screened consumers and delivered the brief intervention where appropriate. Consumers also completed a questionnaire on the process. At three months consumers were telephoned to enable ‘retention’ to be quantified. After completing recruitment, a semi-structured interview was conducted with pharmacists on the process of delivering the intervention, potential improvements and sustainability.

**Results:**

Fifty consumer participants were screened, ten from each pharmacy. There were 28 (57 %) men and 21 (43 %) women with one not responding. Most (67 %) were aged 25–55 years. Their AUDIT scores had a range of 0 to 39 (mean 10.9, SD 9.8) with 11 categorised as ‘hazardous (8–15)’, four as ‘harmful (16–19)’ and eight as ‘probably dependent (20+)’ consumers of alcohol. Reactions to the process of SBI were generally favourable: for example 75 % agreed that it was either appropriate or very appropriate being asked about their alcohol consumption. With respect to follow-up interviews, 23 (46 %) agreed that they could be contacted, including five from the highest AUDIT category. Subsequently 11 (48 %) were contactable at three months. Three of the five non-low risk drinkers had reduced their level of risk over the three months. Ten pharmacists participated in semi-structured telephone interviews. Overall these pharmacists were positive about the intervention and five main themes emerged from the interviews: 1) flexibility applied in recruitment of participants, 2) easiness in use of AUDIT score to facilitate discussions, 3) perceived positive intervention impact, 4) enhanced role of community pharmacists and 5) facilitators and challenges experienced.

**Conclusions:**

Pharmacy-based SBI appears to be acceptable to consumers and feasible for pharmacy staff to deliver. Challenges remain in translating this potential into actual services.

**Electronic supplementary material:**

The online version of this article (doi:10.1186/s12889-016-3805-3) contains supplementary material, which is available to authorized users.

## Background

As primary care providers, community pharmacy staff are often the first point of contact for consumers accessing the health care system [1]. Pharmacists and other pharmacy staff regularly assist consumers with the management of minor, self-limiting symptoms [2, 3]. These symptoms could be associated with inappropriate alcohol use (e.g., indigestion, headaches, sleeping irregularities or ‘hangovers’). Thus, through non-prescription medicine requests, staff can identify consumers who may have risky drinking behaviours and there is potential to educate consumers about alcohol consumption. Pharmacists also have regular contact with consumers through the dispensing of medicines which provides an opportunity to screen and educate consumers about various healthcare related issues including safe drinking guidelines and alcohol-related contra-indications. Additionally, community pharmacists provide a range of primary health care services and interventions [4, 5]. These include medication reviews, disease state management (e.g., mental health and cardiovascular disease management) and lifestyle support such as smoking cessation and weight loss programs [6, 7].

Evidence supports the impact of community pharmacy interventions on consumer health outcomes [8–12]. Randomised and other trials provide proof of the clinical and cost-effectiveness of community pharmacy lipid management interventions in reducing risk factors for coronary heart disease [13, 14]. Pharmacist interventions also assist consumers with smoking cessation [14–17] and many now provide smoking cessation advice as part of normal practice. A scoping review about the role of community pharmacists identified a range of successful services including smoking cessation, healthy eating, lifestyle advice, provision of emergency hormonal contraception, infection control and prevention, promoting cardiovascular health and blood pressure control, and addressing drug abuse [18]. However, the role of Australian community pharmacy in public health promotion has been underutilised [19]. A 2007 review specifically identified a lack of information about the effectiveness of community pharmacy-based services for alcohol misuse, identifying a need to include community pharmacies as part of strategies to address excessive alcohol use [20]. The situation does not appear to have progressed since the review.

The effectiveness of screening and brief intervention (SBI) in reducing alcohol related problems is well established [21, 22] and often incorporates referral for extensive treatment for those with more severe problems (e.g., alcohol dependence) [23]. SBI uses screening instruments such as the 10-item Alcohol Use Disorders Identification Test (AUDIT) [24] or the abbreviated three item AUDIT-C [25, 26] to identify those engaged in ‘risky’ alcohol use who then receive a brief intervention (BI). Typical elements include advice, normative feedback, goal setting, risk reduction strategies, encouraging individual responsibility for change and motivation enhancement [21]. The SBI approach has been recommended for general practices, emergency departments, [27] [28] and recently rural Australian community pharmacies [29].

### Community pharmacy alcohol interventions

A World Health Organisation report on alcohol interventions in primary healthcare emphasised the variety of locations in which alcohol BI can be provided [30]. As primary healthcare providers, community pharmacies are in an ideal situation to complement existing services. However, a survey of New Zealand (NZ) pharmacists showed that pharmacists knowledge of recommended ‘low risk’ drinking limits was poor, although the participants were keen to take on a role in alcohol SBI [31]. This study highlighted the need to provide pharmacy staff with training prior to delivering an alcohol SBI service.

Research in NZ and the United Kingdom (UK) on SBI with problem drinkers indicated that pharmacists considered there was scope for alcohol health promotion in community pharmacy [32, 33]. Participants identified a need for campaigns to raise awareness of risky drinking, appropriate screening tools and pharmacist training whereas barriers to SBI included concerns about offending or alienating consumers, lack of experience or confidence, workforce pressures, privacy and remuneration [31]. Another study conducted in the UK indicated high consumer willingness to participate in SBI and follow-up appointments with the pharmacist to discuss further alcohol use [34]. The researchers conducted a subsequent pre- and post-experimental study involving 26 community pharmacies during which three-quarters of the participants were identified as risky drinkers. Three-month follow-up interviews with people drinking at hazardous levels found that they significantly reduced their consumption and drinking days [35].

These studies show that community pharmacists are willing to deliver SBI provided that pharmacy staff receive training. The above research suggests that SBI in community pharmacy is feasible with positive feedback from consumers. However, there have not been any reported Australian studies on the potential role of community pharmacy in alcohol SBI. Australian community pharmacies need to have a private area in the pharmacy to offer certain government reimbursed services referred to as in-pharmacy medication reviews (MedsCheck and Diabetes MedsCheck) [36] and vaccination services [37]. Australian community pharmacies hence have private or semi-private areas for the pharmacist to conduct certain professional services [38]. This space is also used to discuss confidential and sensitive issues with consumers [39], conduct screening services such as blood pressure measurements and could be used for SBI.

Despite the evidence to support SBI in general practice many general practitioners do not routinely assess patients for risky drinking behaviour or provide alcohol drinking advice to high risk groups [40, 41]. Providing alcohol screening and intervention in community pharmacies will provide an alternative primary healthcare setting to address alcohol misuse and health-related issues. It is therefore timely to introduce and evaluate SBI in Australian community pharmacies.

## Methods

This feasibility study aimed to evaluate an SBI intervention in community pharmacies through assessing 1) the feasibility of recruiting and training pharmacists in SBI techniques, 2) the acceptability of SBI for alcohol use among consumers in pharmacies, 3) process outcomes for pharmacists delivering SBI and 4)’retention’ of consumers at three months.

**Fig. 1:**
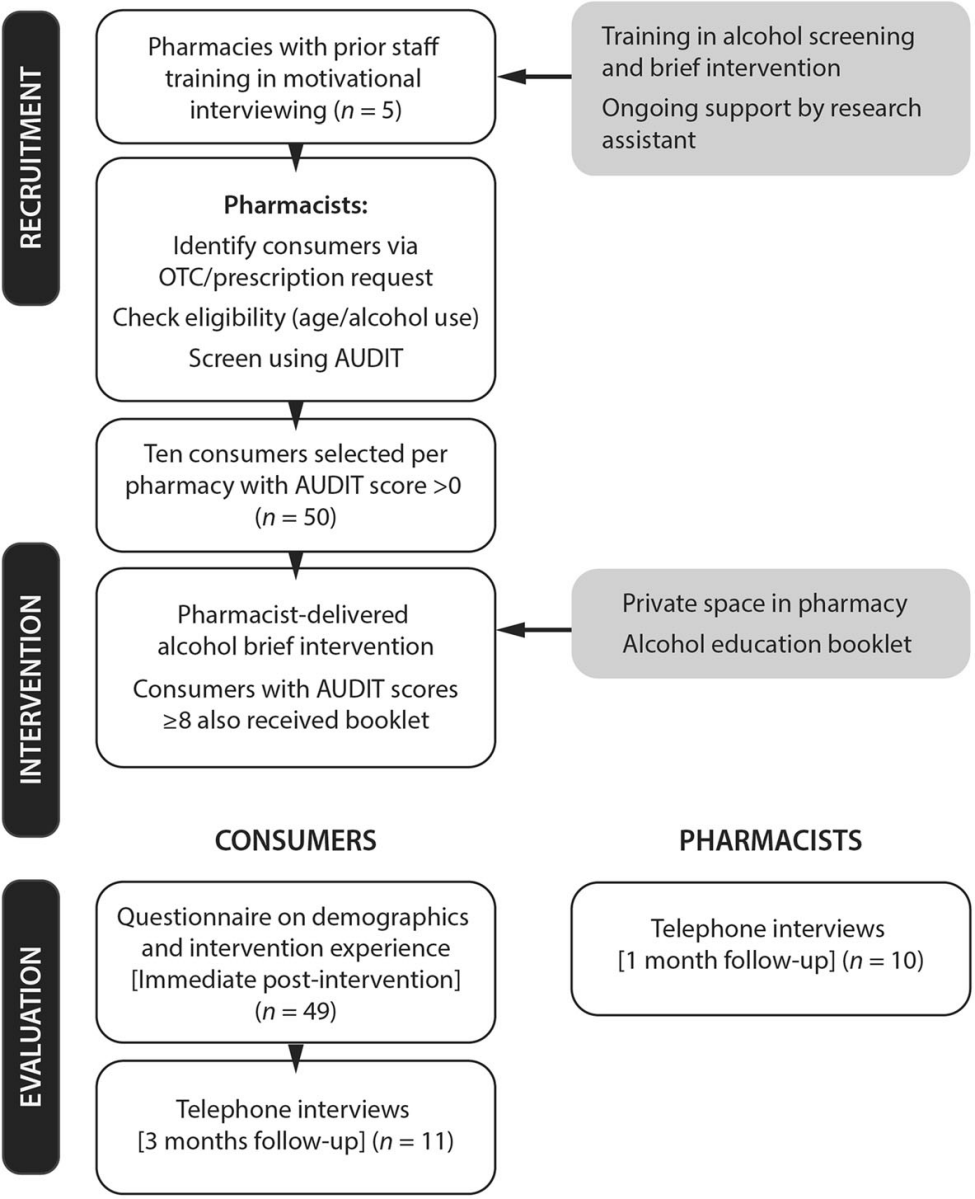
Study flow diagram

This mixed methods cross sectional study involved five community pharmacies in Perth, Western Australia. Quantitative and qualitative strategies (surveys and interviews) were used to collect data. Figure 1 provides an overall flow diagram of the study. This study was approved by the Curtin University Human Research Ethics Committee (PH-16-14).

### Participants

We purposively selected and recruited five metropolitan pharmacies via existing networks: these pharmacies had previously received training in motivational enhancement in an earlier study which focused on increasing the role of community pharmacy staff in providing support and professional services to mental health consumers and carers [42, 43]. The pharmacies were situated in suburbs coded as being in the 6^th^, 5^th^, 4^th^, 3^rd^ and 2^nd^ decile rankings on the Index of Relative Socio-economic Disadvantage (note: the 1^st^ decile is the most disadvantaged) [44]. Thus, the suburbs were generally in the low to middle ranks. Pharmacy managers were contacted by telephone, email or in person to seek their interest and approval to participate in the study. A pharmacy participant information sheet was provided which outlined the study’s background, aim, objectives and method. Once the manager agreed, the pharmacies were visited by the study’s research assistant who was an experienced community pharmacist and was a mentor for pharmacy staff during the aforementioned mental health study and therefore known to the pharmacists [43]. During the visit the research assistant delivered training in alcohol SBI and provided study materials including a recommended flowchart of the process to follow with each consumer (see Additional file 1), consumer questionnaires and alcohol booklets entitled *Here’s To Your Health: A guide to reducing alcohol-related risks and harms* [45]. The research assistant also provided ongoing support to the study pharmacists through regular emails and telephone calls.

The objective was for each pharmacy to recruit 10 consumers (*n* = 50). Consumers were recruited over an eight week period during October to November 2014. Consumers aged 18 or older were eligible, provided that they had sufficient English language skills to give written informed consent. To facilitate recruitment of potential risky alcohol users, consumers requesting certain non-prescription medications relevant to alcohol use such as ‘hang-over cures’, reflux/heartburn medications and sleep aids, were approached. Consumers who presented with prescriptions for certain chronic conditions that require diet modification were also approached, for example those with peptic ulcer disease or diabetes, as well as those with medications contra-indicated with alcohol such as certain antibiotics that can cause a disulfiram-type reaction (e.g., flushing, sweating, nausea, vomiting, palpitations, headache, dyspnoea, chest pain, hypotension, cardiovascular collapse, seizures, arrhythmias) or medications with increased falls risk due to increased drowsiness such as certain anti-psychotics, hypnotics and opioid analgesics [46]. These consumers were initially targeted as the non-prescription request or dispensing provided the pharmacist with an opportunity to discuss lifestyle interventions and management, including alcohol use, and to assess their overall wellbeing. Pharmacists therefore had to use their professional judgement in deciding which consumers to approach.

### Intervention

Consumers who agreed were then taken to a private space in the pharmacy to complete the 10-item AUDIT [47] to determine alcohol use. The reference period for the AUDIT is the previous 12 months and scores range from 0–40. Those scoring 0 are non-consumers, 1–7 are categorised as ‘low risk’, 8–15 as ‘hazardous’, 16–19 and ‘harmful’ and 20 or more as ‘probably dependent’ consumers. Consumers with scores ≥1 were eligible for this feasibility study.

Pharmacists then completed the scoring with the consumers; Table 1 provides a summary of the pharmacists’ proposed responses, depending on the consumer’s score. The pharmacists talked to the consumers about their scores and used motivational interviewing techniques to facilitate behaviour change [48]. Consumers with a score ≥8 also received the alcohol booklets.

**Table 1:** Pharmacist recommended response according to AUDIT score

Score	Recommended response
0	Thank them for their time (they were not eligible for the study)
1-7	*“From your answers, it appears that you are at low risk of experiencing alcohol-related problems if you continue to drink moderately”.* (If applicable tell them about lifetime/single occasion risk if appropriate). *“Finally, unless you have any questions, could you fill out this short anonymous survey please?”*
8-15	*“looking at the results of the AUDIT it appears that you may be at risk of experiencing alcohol-related problems if you continue to drink at your current levels; I would like to take a few minutes to talk with you about it”.* (Go to “Here’s To Your Health” booklet information)
16-19	“*looking at the results of the AUDIT, you may be experiencing alcohol-related problems from your current drinking. I would like to take a few minutes to talk with you about it*”. (Go to “Here’s To Your Health” booklet information)
20+	*“I should emphasize that the AUDIT doesn’t give a diagnosis, but on the basis of your results I would recommend that you see your doctor or a specialist as soon as possible to discuss your use of alcohol as you appear to be exceeding safe limits and it may already have caused you harm. I would like to take a few minutes to talk with you about it”.* (Go to “Here’s To Your Health” booklet information)

Following the intervention consumers were requested to complete a questionnaire to obtain basic demographic information as well as their:

- Opinions about their alcohol use,
- General self-rated health item from the Medical Outcomes Study [49],
- Opinions on the pharmacy alcohol screening service and whether that would affect future alcohol use,
- Experience of the service, appropriateness of the delivery of the intervention (e.g., privacy, time constraints) and acceptability of being approached and screened for alcohol use in the pharmacy, according to 12 5-point Likert scale questions,
- Consultation with the pharmacist on eight 5-point Likert scale questions, and
- Use of the internet for health-related information.

Consumers then received a $10 gift card for their time commitment and participation in the study. Consumers were also asked if they could be contacted after a three-month period to assess whether there was any change in alcohol use. Consenting consumers had to provide a contact telephone number and a preferred time to be contacted. At three months, consenting consumers were telephoned to collect current alcohol use data (AUDIT score) to enable ‘retention’ to be quantified.

### Pharmacist interviews

At the end of the consumer recruitment period, a semi-structured interview was conducted with pharmacists on the process of delivering the intervention, potential improvements and sustainability. An interview tool was developed considering the literature that consisted of 20 open-ended questions with prompts. Questions were categorised to cover their:

1. Experiences with:
  asking consumers to complete the AUDIT tool,
  screening consumers’ alcohol use through interpreting completed AUDIT forms,
  provision of consumer feedback following interpretation of the AUDIT score,
  asking consumers to complete the anonymous survey, and
2. Opinions about:
  the broader impact of participating in the study, and
  provision of alcohol SBI services in pharmacies.

Pharmacists provided consent to being interviewed and received a $30 gift card as a small token of appreciation for their time. Interviews were audio recorded.

Quantitative data collected were analysed using the statistical software program SPSS version 22 [50]. Given the low cell count in many of the frequency tables, statistical analysis was not appropriate in most instances, so the results are primarily descriptive. Audio recorded qualitative data was transcribed verbatim and was prepared for in-depth analysis by 1) conducting quality checks on a sample of the transcribed interviews (a researcher not involved in conducting or transcribing the interview listened to the recording whilst reading over the transcript in order to check for accuracy) and 2) removing identifiable information from transcripts (such as person, place and business names). The data was subsequently analysed for common themes or categories through the general inductive approach [51]. Transcripts were read and re-read to gain an understanding of the broad issues relative to the key evaluation questions. Specific themes were subsequently developed and supporting quotations documented under each theme category which captured core messages.

## Results

Five metropolitan pharmacies were enrolled and they screened 50 consumer participants with 10 from each pharmacy. Recruitment time ranged from 3 days to 8 weeks.

### Intervention analysis

There were 28 (57 %) men and 21 (43 %) women (missing *n* = 1). The modal age group was 45–54 years (*n* = 12) with 25–34 years (*n* = 11) and 35–44 years (*n* = 10) the next largest groups. The mean AUDIT score was 10.9 (SD 9.8) with a range of 1–39. Table 2 shows key measures by AUDIT categories. Most participants rated their health as ‘good’ (*n* = 20, 40 %) with a further 13 nominating ‘very good’ or ‘excellent’ (*n* = 3). The health rating did not appear to be related to their AUDIT category. Most (75 %) of those in the highest AUDIT category identified their present level of alcohol use as harmful to their health compared with 11 % in the lowest category.

**Table 2:** Consumers’ demographic data and alcohol use

	AUDIT Category			
	‘Low risk’ (*n* = 27)	‘Hazardous’ (*n* = 11)	‘Harmful’ (*n* = 4)	‘Dependent’ (*n* = 8)
Sex				
female n (%)	13 (48)	4 (40)	1 (25)	3 (37)
male n (%)	14 (52)	6 (60)	3 (75)	5 (63)
Age group				
55+	5 (19)	1 (10)	1 (25)	2 (25)
45–54	8 (30)	2 (20)	1 (25)	1 (12)
35–44	4 (15)	3 (30)	1 (25)	2 (25)
18–34	10 (37)	4 (40)	1 (25)	3 (38)
Self-rated health				
poor/fair n (%)	5 (30)	1 (9)	2 (50)	3 (37)
good n (%)	9 (33)	6 (55)	2 (50)	3 (37)
v. good/excellent n (%)	10 (37)	4 (36)	–	2 (25)
Self-rated alcohol harm				
yes n (%)	3 (11)	6 (55)	2 (50)	6 (75)
no n (%)	23 (85)	4 (36)	2 (50)	2 (25)
don’t know n (%)	1 (4)	1 (9)	–	–
N^o^ GP visits				
mean (SD)	3.0 (4.1)	1.6 (2.1)	1.5 (1.7)	1.9 (1.5)

*AUDIT* Alcohol use disorders identification test, *GP* general practitioner

Table 3 presents the results of respondents’ experience of the process. Inspection of the distributions did not suggest that rating were associated with participants’ alcohol use. For example, 87 % of the highest AUDIT category either agreed or strongly agreed with item 3 (“comfortable about discussing my alcohol…”) compared with 96 % of the lowest category. Similarly, 50 % of the highest category agreed or strongly agreed with item 12 (“…I would use this pharmacy more…”) compared with 41 % of the lowest category. Participants were also asked about their options on having alcohol screening services in community pharmacies. As with the other measures, these did not appear to be related to their AUDIT category and were generally supportive (Table 4).

**Table 3:** Experiences of the alcohol assessment process^a^

Item	Strongly agree	Agree	Neither agree/disagree	Disagree	Strongly disagree
Easy to complete	28 (58)	17 (35)	3 (6)		
Screening was discreet	31 (62)	15 (30)	4 (8)		
Comfortable to discuss	31 (62)	14 (28)	4 (8)	1 (2)	
Embarrassing being asked	2 (4)	5 (10)	13 (26)	15 (30)	15 (30)
Confidential manner	27 (55)	15 (31)	5 (10)	2 (4)	
Happy to discuss alcohol in the future	26 (52)	19 (38)	4 (8)	1 (2)	
Prefer to discuss with my GP	1 (2)	10 (20)	24 (48)	8 (16)	7 (14)
Leaflet was helpful	10 (21)	27 (56)	10 (21)	–	1 (2)
Prefer not to discuss	2 (4)	5 (10	13 (26)	22 (44)	8 (16)
Regular customer of pharmacy	24 (49)	19 (39)	3 (6)	2 (4)	1 (2)
Would now use this pharmacy less	1 (2)	2 (4)	8 (16)	14 (29)	24 (49)
Would now use this pharmacy more	10 (20)	13 (26)	23 (46)	1 (2)	3 (6)

^a^See Additional file 1 for the full wording of items

**Table 4:** Opinions on the alcohol screening service in community pharmacies

	AUDIT Category			
	‘Low risk’ (*n* = 27)	‘Hazardous’ (*n* = 11)	‘Harmful’ (*n* = 4)	‘Dependent’ (*n* = 8)
How appropriate				
V. app/appropriate n (%)	20 (80)	6 (9)	3 (75)	7 (88)
neither n (%)	4 (16)	4 (36)	–	–
V. inapp/inappropriate n (%)	1 (4)	6 (55)	1 (25)	1 (12)
Amount consumed				
increase n (%)	–	–	1 (25)	–
no effect n (%)	20 (77)	2 (20)	3 (75)	3 (60)
reduce n (%)	6 (23)	8 (80)	–	2 (40)
How helpful was this service				
V helpful/helpful n (%)	12 (48)	11 (100)	1 (25)	5 (71)
neither n (%)	12 (48)	–	2 (50)	2 (29)
V. unhelpful/unhelpful n (%)	1 (4)	–	1 (25)	–

### Follow-up

Out of the 50 participants, 23 gave permission to receive a follow-up telephone call at three months, including five of the eight from the highest AUDIT category. This resulted in 11 (48 %) completed interviews, seven calls went to voice-mail with no returned calls, three made contact with the person who asked to be called back but was not subsequently obtainable, one number was unobtainable, and one was the wrong number/person did not remember the survey. Of the six low risk drinkers at baseline, all were still in the same category at three months. The one hazardous user had moved to probably dependent. Two of the probably dependent users were now classified as low risk, one as hazardous and one remained as probably dependent. Thus, three of five non-low risk drinkers had reduced their level of risk.

### Pharmacist interview analysis

Ten pharmacists participated in semi-structured telephone interviews (six males, four females) during December 2014, two from each of the participating pharmacies. Interviews were on average 18.7 min (min. 6.20 min, max. 35.48 min). Five main themes emerged from the data namely:

- Flexibility applied in recruitment of participants
- Easiness in use of AUDIT score to facilitate discussions
- Perceived positive intervention impact
- Enhanced role of community pharmacists
- Facilitators and challenges experienced

These themes are presented with illustrative quotations.

### Flexibility applied in recruitment of participants

Overall comments were that recruitment of consumers was fairly straight forward. The pharmacists used flexible strategies to approach consumers with whom they already had relationships i.e., consumers with certain chronic conditions such as diabetes or hypertension, or on the opioid substitution program. More generic approaches were also followed such as prompting consumers to participate while the consumers were waiting for their prescriptions to be dispensed and some pharmacists targeted consumers who requested specific over-the-counter medicines on Sundays or those with insomnia:

*“But also my other target group was people buying pain killers or when they would come over on a Sunday morning buying Gastrolyte® or you know, Powerade®. They’ve had a big night out…* “(P5)

*“I’d probably go for the ones that have trouble sleeping because often people use alcohol either as a pain killer or to help them sleep”.* (P5)

The pharmacists therefore used multiple strategies that included approaching consumers with whom they had already had a relationship as well as targeting consumers on or requesting specific medicines.

### Easiness in use of AUDIT score to facilitate discussions

The pharmacists reported that it was relatively easy to work through the AUDIT questions and to talk to consumers about their social habits. They provided low risk consumers with feedback and education and generally these consumers were receptive to receiving information. More structured approaches were used to provide feedback to consumers with scores that indicated they were at risk of alcohol-related harm, the feedback focused on the impact on these consumers’ health:

*… it did open up a discussion in terms of their general health and how it’s going to affect them and how it’s effecting their liver, and other conditions or other parts of their body”.* (P1)

Most pharmacists were of the opinion that those consumers with alcohol-related problems were already aware of them:

*“I didn’t find it challenging at all, like people that obviously like scored really high scores, knew they had a problem. They knew that, they know you know, it’s not as if they were quite surprised by it. I think if you’ve got a drinking problem you generally know about it”.* (P4)

Some reported that it was more challenging to provide feedback to consumers who were at high risk due to their alcohol consumption or consumers on specific medicines:

*“… they will be a bit hesitant if they drink regularly or a very heavy alcoholic”.* (P6)

*“If they were drinking once in a while they were quite happy to fill that in for us, to be honest. If they are on some certain medications they don’t really like to disclose their information about their drinking”.* (P6)

However, the tool provided an opportunity to initiate discussions about the risks involved:

*“I guess it is something that, putting it down on paper they saw how bad it could have been, so yes we had a few looks at the alcohol [consumption] and they would kind of laugh it off and be like moving on until it got to some of the more serious questions”.* (P2)

### Perceived positive intervention impact

All pharmacists agreed that working through the AUDIT scores with the consumers provided an opportunity to talk about alcohol use:

*“… with this I found that a lot of people who do drink alcohol without realising that they’re possibly addicted to the drug potentially, and I think doing the alcohol study and the screening process it sort of, it makes the invisible visible. It brings that out … It allows the person to evaluate their own condition more objectively*. … *It will definitely allow them to think about what they’re doing and their whole lifestyle so it may have an implication on their health, eating habits as well because often alcohol is associated with going out”.* (P5)

*“… putting it on paper how much it could be affecting their life”.* (P2)

*“… they found it to be helpful especially if on the higher risk about how they can go about, even monitoring what they are taking, how much they are drinking. So I believe it has had … some positive impact on some of them. I think some of them as well would like to [participate] further, if there were more studies they would like to participate”.* (P9)

The pharmacists found the use of the alcohol booklets particularly a helpful resource:

*“… they were well received and some information that I give them was new. So this gave them more information on alcohol intake.... Some of them you could see that they were happy, that it was new information for them: ‘Hey, I know where I’m at and I should cut back.’”* (P9)

*“I think that was a massive positive, that we were able to help one out of those ten that did want a follow up. And the information was invaluable to them*”. (P5)

One pharmacist reported that the intervention was very motivating to one consumer who was already in treatment and as a result of the intervention realised his/her progress:

*“One patient in particular, to put it on paper how bad it was because he was in a recovery stage, he had actually been through quite a lot that I didn’t know about. So he was opening up a bit about it, but it did make him realise how far he’s come. He was at the point of alcohol abuse at that stage and couldn’t live with it, so to have him put it down on paper and to understand that he was going through the whole process with his doctor and still, realise that he was at a better stage having looked through the questions”.* (P2)

There was also a report of facilitating hospitalisation of a consumer through the intervention:

*“I had one guy who was very severe, if we’re looking at the other end of the spectrum I think he had symptoms of pancreatitis and that was a referral to a hospital, so that was at that end as well and I had to call the ambulance in that day as well because he was in that much pain and he starts his day with a cup of wine at 5 am, he was very severe”.* (P5)

### Enhanced role of community pharmacists

All of the pharmacists agreed that their involvement in the project had positively contributed to the professional services image of the pharmacy and was good for business. It also made them and the other pharmacists more proactive in talking to consumers about alcohol use:

*“… it made the pharmacists to be more aware and to be more proactive as well when they approach customers”.* (P7)

*“… it’s just good to get myself and pharmacists sort of in the habit of, I mean this is a very much service orientated and not product centric … it was just good and it sort of helped us to increase our skills”.* (P3)

In addition it also assisted with building relationships with consumers and expanding the scope of services provided by community pharmacists:

*“I suppose it allowed us to build that relationship with that person as opposed to just asking them, we’re going beyond that. … that shows the customer that you’re actually interested in their health and not just there to do a task*”. (P5)

*“… generally only had positive responses so that was because they were willing to do it, probably shows that they do see the community pharmacy being part of the healthcare team”.* (P10)

Participation in the alcohol SBI provided pharmacists with an opportunity to link alcohol use with the management of chronic health conditions:

*“… some people that were on high risk obviously and moderate risk we spoke to them if they had any blood pressure problems or, you know you usually have the medication next to you because you have dispensed something and have a little bit of a discussion how reducing alcohol intake can reduce blood pressure”.* (P8)

One of the pharmacists specifically commented that participation in the study provided an opportunity to facilitate building of better relationships and organise follow-up visits:

*“… it would be looking at how we can help them to curb their alcohol addiction because we ask them if they were to go off alcohol for a few days how would they feel about that, they’re the ones that you know they need the help and often I would ask them to come back and see me. I had a couple that have come back … it’s a slow work in progress because these sorts of things don’t happen overnight. … I would talk to them about other services that are available for them as well, that were outlined in the book and I’d give them a book as well and probably highlight a few services in there”.* (P5)

### Facilitators and challenges experienced

Table 5 provides selected quotations related to facilitators and challenges. The pharmacists responded that the study paperwork and the AUDIT tool were straight-forward and easy to implement. Using a flexible approach and tailoring the intervention according to the needs of each individual consumer also facilitated uptake.

**Table 5:** Facilitators and challenges to provision of alcohol SBI services

Facilitators	
Straight-forward tool	*“.... being a really straight forward screening test works really well*”. (P9)
Flexible approach	*“… we just leave the questionnaire there for the customers to do it, what I found worked was I would have a generalised chat with the customers and then I would tick them off as they’re talking,...”.* (P5)
Challenges	
Time	*“… if there were any challenges it would be time because if we have many customers then it’s a bit tricky*”. (P9) *“Time management would be the main barrier mainly”.* (P1)
Privacy	*“… maintaining that level of privacy while you’re discussing very personal questions, that was probably a big challenge”.* (P5) *“… some of the customers might think that we are actually invading their privacy if we ask too much about alcohol drinking so we try to maintain and retain the relationship with the customers*”. (P6)

Specific challenges reported were lack of time to provide the service, especially during busy periods. Another challenge was the need for increased consumer privacy in the pharmacy and consumers’ concerns about sharing sensitive private information.

## Discussion

This study was conducted to investigate the feasibly of delivering alcohol screening and brief interventions in community pharmacy in Australia. Overall, the participating pharmacists regarded the screening process as an appropriate activity for pharmacists to conduct and saw it as a useful and positive means of engaging with consumers. Similarly, the consumer feedback was largely supportive of pharmacists asking about their use of alcohol and providing information and advice.

The cross-tabulation of the AUDIT categories and self-reports of the likely impact of current level of alcohol use on their health, suggests that many consumers are cognisant of alcohol-related harms from their drinking. However, this was not reflected in their general self-rated health, as on this dimension, ratings were comparable across AUDIT categories. The 2013 National Drug Strategy Household Survey similarly showed that risky drinkers were less aware of the number of standard drinks an adult could drink before putting their health at risk [52]. It is worth noting that although self-rated health has been found to be associated with mortality, the processes underpinning this relationship between this subjective rating and biological outcomes are unclear [53]. However, it seems likely that in this instance respondents were aware of a broader range of factors than just alcohol and its effect on their health.

Almost all of the consumers indicated that it was easy to complete the AUDIT tool (93 %), the screening was conducted in a discreet manner (92 %) and they would be happy to discuss alcohol use with a pharmacist in the future (90 %). These results support an UK study that showed positive opinions about the desirability and feasibility of pharmacy-based alcohol services from relevant stakeholders, including members of the public [54]. Specific services perceived as appropriate by consumers in the UK study were to support people to reduce drinking, provision of written advice and information about other services, and referral into other services [54]. Another UK study that evaluated feasibility and acceptability of alcohol SBI in community pharmacies for women accessing emergency contraception likewise reported that consumers were not embarrassed, were happy to talk to the pharmacist and be given advice, and felt that the pharmacist was a suitable person to provide alcohol SBI [55].

Ten percent of consumers in the current study were neutral and 4 % disagreed that the service was provided in a confidential manner. This indicates that there is a need for some pharmacies to improve privacy and confidentiality aspects, which has also been identified as barriers in other community pharmacy alcohol screening studies [54, 56]. An Australian study that focused on privacy and confidentiality needs of mental health consumers and carers highlighted a need for increased staff training about the importance of privacy and confidentiality, workflow models to facilitate private discussions and processes and procedures to ensure confidentiality of documentation [57]. Community pharmacies providing alcohol SBI services will similarly need to ensure that these aspects are well managed.

The pharmacists who participated in the alcohol SBI provided positive feedback and highlighted that flexibility in approaching and working with consumers worked well. In some cases consumers who had certain chronic conditions, such as diabetes, were targeted and this provided an opportunity to discuss the overall management of the chronic condition. In other cases consumers were targeted who requested specific non-prescription medicines, such as medicines for heartburn. The study provided an opportunity for the pharmacists to discuss health-related issues with consumers which in turn enhanced therapeutic relationships between the pharmacists and the consumers. Australian research has demonstrated that the public see pharmacists as trustworthy medicine experts, who are reliable advisors on health matters and with collaborative relationships with the medical profession [5, 6]. Various other studies indeed support the role of community pharmacists in health promotion and disease prevention activities [58], including activities to increase consumers’ alcohol awareness [59]. Challenges experienced by the pharmacists included time constraints and lack of privacy, obstacles which had also been reported in other studies, as well as a need for appropriate remuneration for services [56, 60]. The need for appropriate remuneration for the delivery of professional pharmacy services has indeed been highlighted over recent years and the Department of Health is, at the time of writing, conducting an independent national Review of Pharmacy Remuneration and Regulation following the release of a Discussion Paper in July 2016 [61].

The extensive distribution of community pharmacies provides accessible health care and consumers can visit a pharmacy without an appointment, speak with a health professional almost immediately without incurring a cost, and retain a high level of control over the extent of their engagement with the staff [5]. This presents a unique opportunity for Australian community pharmacists and pharmacy staff members to discuss health related concerns, such as alcohol related illness and relevant social issues with consumers, provide information and facilitate referrals. This feasibility study provided some baseline data about alcohol SBI services provided in community pharmacies with helpful feedback from consumers and pharmacists and perceived positive outcomes. The results from this study could be used to inform the development of future alcohol services in community pharmacies.

One of the limitations of the study is the extent to which the findings will generalise to other pharmacies, in particular, those in more affluent areas and regional and remote locations. Those involved had already collaborated on another project about the provision of mental health services in community pharmacies with a member of the research team. Thus, they represent pharmacists who were interested and engaged with research and are potentially in pharmacies where this is a valued activity and the workload permits this level of interaction with customers. Additionally the pharmacists had ongoing support from the research assistant which will be challenging to follow in a larger scale study. Although this was a feasibility study, we provided a small payment for the time taken to both consumer participants and the pharmacists for their exit interviews. The latter were in excess of any activity with customers and hence had no prospect of remuneration arising from customer loyalty or satisfaction. Similarly, there was no benefit to customers from completing the research surveys unlike the interaction with their pharmacist which could have a health benefit.

### Next steps

The effectiveness of brief alcohol interventions has been demonstrated in primary care settings [21, 62] and there is support for broader health promotion including alcohol screening in pharmacies [31, 32, 63]. We recommend that the focus should now be on maximising their impact, translation of research findings and practice implementation. The cohort of pharmacists in this study had received training in motivation enhancement techniques as part of an earlier study [43]. While face-to-face training is effective and is cheap initially, this approach inhibits further dissemination. There are extensive resources on motivational techniques and recently the Western Australian Department of Health has developed alcohol brief intervention training [64]. However, additional material specifically focused on the pharmacy setting and scenarios could be beneficial and help to increase the confidence of pharmacists to address alcohol use and other issues with their customers. Collaboration with pharmacy professional organisations namely the Pharmaceutical Society of Australia and the Pharmacy Guild of Australia would allow widespread dissemination.

Two main challenges were identified by the pharmacists – time and privacy. Without clear financial incentives, screening and brief intervention cannot be expected to be undertaken during busy times. Nevertheless, there may be low intensity alternatives that could be given to anyone receiving prescription or other medications contra-indicated for alcohol use. It should be standard practice to ask about alcohol when dispensing these products and all affirmative replies could receive a card with a web-address to an online brief intervention [65] or card with the short form AUDIT with advice – this can be summarized on one A4 page [45].

High workload periods also impact on privacy for customers, increasing the need for an area where personal questions can be asked. However, the necessity of such a space to provide professional services [38] such as MedsCheck, Diabetes MedsCheck [36] is an incentive for pharmacists to ensure that they have an appropriate area available.

## Conclusions

The wide distribution and easy access to community pharmacies make them an ideal venue for reaching a substantial portion of the population to deliver health interventions. However, the lack of funding to support SBI for alcohol use problems is a significant impediment to the deployment of this service. Including alcohol SBI within existing programs such as in-pharmacy medication reviews would provide an initial option for funding and target a group where enquires about alcohol use are relevant due to the potential use of multiple medications.

## Additional file

Additional file 1: Script and Flow Chart. Recommended script and flow chart used by participating pharmacists to approach consumers and explain the SBI process. (DOCX 82 kb)

## Abbreviations

AUDIT: Alcohol use disorders identification test
BI: Brief intervention
NZ: New Zealand
SBI: Screening and brief intervention
UK: United Kingdom
