# Supplementary material for: ‘Making the invisible visible’ through alcohol screening and brief intervention in community pharmacies: an Australian feasibility study
Source: BMC Public Health. 2016 Nov 8;16:1141. doi: 10.1186/s12889-016-3805-3 (PMC5101702; doi:10.1186/s12889-016-3805-3)
Supplement: Additional file 1: — Script and Flow Chart. Recommended script and flow chart used by participating pharmacists to approach consumers and explain the SBI process. (DOCX 82 kb) [file 12889_2016_3805_MOESM1_ESM.docx]

**PHARMACY ALCOHOL SCREENING STUDY**

**Script & Flow chart**

*I’m working with Curtin University on project looking at alcohol use in the general community. We are doing a survey on whether or not people think that a pharmacist can provide useful information on the safe use of alcohol, particularly in combination with medicines. Can I talk to you for a few minutes about your use of alcohol? Just to make sure you are eligible for the study,*

- *are you aged 18 or older?*
- *have you drunk any alcohol in the last year?*

*Thank you. Because this is a research project, I need to get your written consent. This page provides a description of the study. After you have read this, if you are happy to go ahead, could you sign the consent form.*

PROVIDE INFORMATION SHEET & CONSENT FORM

*Would you like to come into the consultation area so that we can talk in private? I’m just going to ask you a few questions about your use of alcohol.*

PROVIDE A COPY OF THE AUDIT

(sum the AUDIT scores)

0 – thank them for their time (they weren’t eligible for the study.)

1-7 they are in the low risk range on the AUDIT (but check NHMRC low risk guidelines against their AUDIT answers)

- Low-risk drinking for lifetime risk: no more than 2 standard drinks on any day (check AUDIT Q2)
- Low-risk from a single occasion to reduce risk of injury / accidental death – no more than 4 standard drinks on any one day (check AUDIT Q3)
- High-risk groups e.g. planning pregnancy/ pregnant/ breastfeeding no alcohol: medication interaction: anxiety/depression/PTSD: health conditions (heart disease, high-blood pressure, diabetes, hepatitis, pancreatitis)

*“From your answers, it appears that you are at low risk of experiencing alcohol-related problems if you continue to drink moderately.”*

(If applicable tell them about lifetime / single occasion risk if exceeded).

*“Finally, unless you have any questions, could you fill out this short anonymous survey please?”*

8-15 *“looking at the results of the AUDIT it appears that you may be at risk of experiencing alcohol-related problems if you continue to drink at your current levels; I would like to take a few minutes to talk with you about it.”* (Go to “Here’s To Your Health” booklet information)

16-19 “*looking at the results of the AUDIT, you may be experiencing alcohol-related problems from your current drinking. I would like to take a few minutes to talk with you about it*.” (Go to “Here’s To Your Health” booklet information)

20+ *“I should emphasize that the AUDIT doesn’t give a diagnosis, but on the basis of your results I would recommend that you see your doctor or a specialist as soon as possible to discuss your use of alcohol as you appear to be exceeding safe limits and it may already have caused you harm. I would like to take a few minutes to talk with you about it”.* (Go to “Here’s To Your Health” booklet information)

HERE’S TO YOUR HEALTH BOOKLET

8-15 & 16-19

The “Here’s to your health” booklet will help you decide if you should change your drinking and how to go about it if you do decide to change. A good way to get started is first to think about the things that you enjoy about drinking (pg 18) such as socializing or to relax. Then have a think about the things that aren’t so good about drinking (pg 19) such as the cost or hangovers. Page 22 helps you to summarize your thoughts on if you want to change your drinking.

If you do decide to change, setting yourself a clear goal is very important (pg 24). The next section of the book provides useful information on identifying people who can help you (your support team), how to deal with high risk situation and ways to stay at low-risk. It also provides a free call number if you want any further help (1800 198 024).

*“Finally, unless you have any questions, could you fill out this short anonymous survey please?”*

20+

Because some people who score highly on the AUDIT can have adverse reactions if they stop drinking suddenly, we suggest that you either speak to your own doctor or call the helpline (pg 39 – free call 1800 198 024) before you stop drinking. However, the “Here’s to your health” booklet can help to get you started on thinking about how to change your use of alcohol.

*“Finally, unless you have any questions, could you fill out this short anonymous survey please?”*

**PROVIDE BOOKLET TO THOSE SCORING >7**

**PROVIDE PHARMACY CLIENT QUESTIONNAIRE,**

**Put the completed AUDIT form in envelope and give gift voucher**

**Flowchart**

Identify customer via OTC / prescription request

↓

Check eligibility (age/ alcohol use)

↓

Information sheet and sign consent

↓

AUDIT

↓

Check score and provide appropriate advice and booklet

↓

Pharmacy client questionnaire, envelope & voucher

↓

Sign payment log
